# Necrotizing Craniocervical Soft Tissue Infections: Clinical Experience and Personal Considerations 

**DOI:** 10.1155/2012/489638

**Published:** 2012-12-04

**Authors:** Stefania Gallo, Apostolos Karligkiotis, Riccardo Lenzi, Paolo Castelnuovo, Iacopo Dallan

**Affiliations:** ^1^Department of Otorhinolaryngology, University of Insubria, Varese, Ospedale di Circolo e Fondazione Macchi, Via Guicciardini 9, 21100 Varese, Italy; ^2^Department of Otorhinolaryngology, University of Pisa, Pisa, Azienda Ospedaliero Universitaria Pisana, Via Paradisa 2, 56100 Pisa, Italy

## Abstract

Necrotizing cervical soft tissue infections (NCSTIs) are devastating uncommon clinical entities that are often life threatening. We report two patients suffering from NCSTI and treated at our institution. Diagnosis of NCSTI has been confirmed histologically and surgically. Both patients were managed with very aggressive treatment (medical and surgical) and survived with minimal morbidity. Early diagnosis and aggressive, multimodality treatment can reduce mortality and morbidity rates. Thoracic and mediastinal involvement requires appropriate management. A strong clinical suspicion remains one of the most important aspects of the management of such shattering conditions.

## 1. Introduction

Necrotizing cervical soft tissue infections (NCSTIs) are uncommon clinical entities, often life threatening. More than ten years ago, Maisel and Karlen stated that necrotizing soft tissue infections of the neck are difficult to categorize [1]. In fact, distinct entities can be recognized in this heterogeneous group and, among them, craniocervical necrotizing fasciitis are the most common. Most cases originate from odontogenic infections [2, 3], but there are reports of cases originating from the upper airways [4, 5]. Iatrogenic cases have also been described [6]. These dramatic conditions often show with fatal complications, such as descending mediastinitis and thoracopleural empyema.

We report our experience with two patients affected by NCSTI and stress the importance of an early and extensive surgical treatment.

## 2. Case Reports

### 2.1. Case 1

**Figure 1:**
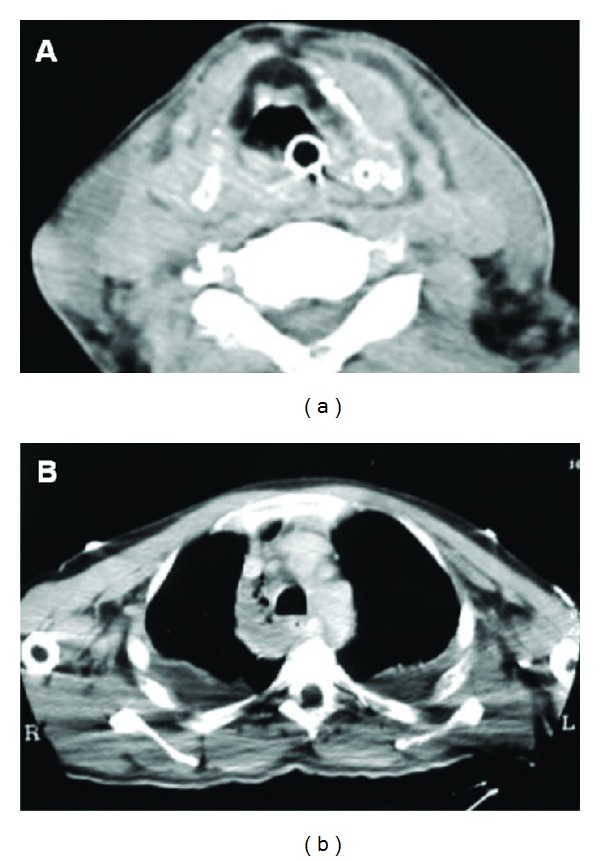
Patient number 1—CT images. (a) Area of effusion of liquid mixed with gas is visible between muscle planes in the cervical area. (b) Expansion of mediastinum due to presence of liquid and gas can be easily observed and a pleural effusion is clearly visible bilaterally.

**Figure 2:**
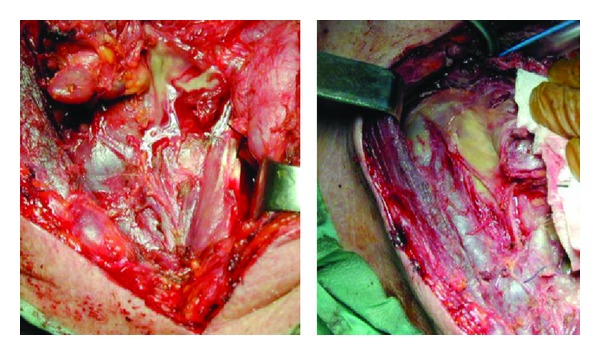
Patient number 1—surgical images. Yellow purulent secretions between muscular planes can be easily observed.

A 57-year-old nonsmoker nondrinker male was admitted to the emergency department with clinical signs of pharyngotonsillitis associated with poor health conditions and treated with antibiotics (ceftriaxone and ciprofloxacin). Unfortunately, his condition precipitated and signs of cardiorespiratory failure arose with a need for orotracheal intubation and aggressive medical therapy (teicoplanin, gentamicin, cardiotonics, steroids, and volume expanders). However, his general health deteriorated and the hepatorenal and cardiorespiratory conditions worsened with an evidence of elevated neutrophils. An objective evaluation of the neck revealed the presence of an underlying area in the anterior and lateral cervical areas with signs of subcutaneous phlogosis but without signs of colliquation and crepitation. Oedema and diffuse hyperaemia of the supraglottic area were seen on the endoscopic evaluation. A computed tomography (CT) of the neck and chest revealed the presence of small pools of liquid/gaseous material in the entire cervical area with considerable detachment of the muscle planes, without a clear evidence of abscess, and an expansion of the mediastinum with pleural effusion/empyema (Figure 1). A left neck dissection plus anterior and posterior drainage of the mediastinum via cervicotomy were performed with an evidence of yellow purulent secretions between the muscular planes (Figure 2); the necrotic tissue was excised and the surrounding areas were removed until healthy bleeding tissue was reached. All the vital structures, including the internal jugular vein and the accessory spinal nerve, were preserved. The flap was repositioned without stitching the wound and the next day a right neck dissection plus a revision of the left one was performed. Drains were inserted in the neck and in the mediastinum and lavages were performed several times a day with antibiotic solutions (rifampicin). A conventional tracheotomy was performed in order to make easier the management of bronchial secretions and breathing. Histologic analysis demonstrated necrotic areas involving the connective tissues and partially the muscular tissues. Bacteriologic analysis of the surgical specimen revealed a composite flora: *Streptococcus viridans*, *Staphylococcus epidermidis,* and the *Bacteroides buccae* anaerobe. After these procedures, the patient's condition, although critical, gradually improved and he was discharged in good health three months after his admission with no significant complications.

### 2.2. Case 2

**Figure 3:**
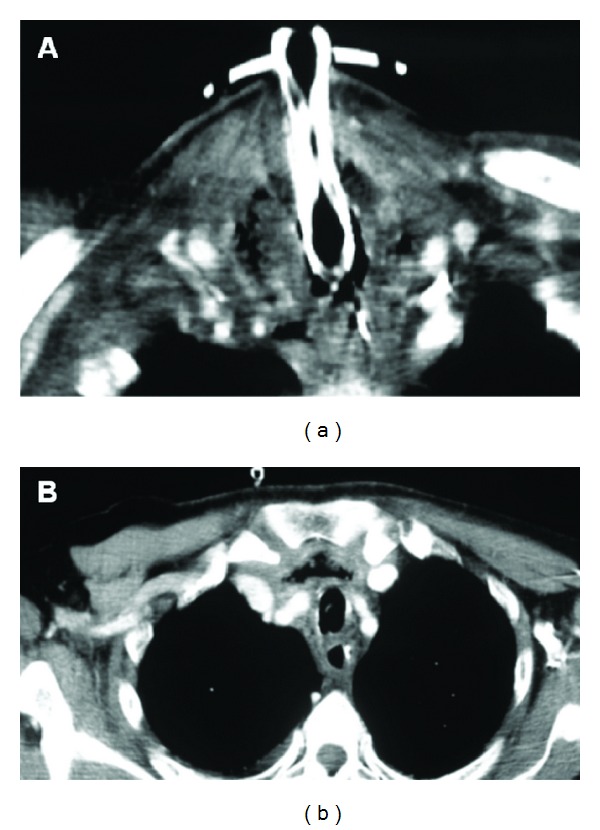
Patient number 2—CT images. (a) Pools of liquid/gaseous material in the neck planes, around the trachea, can be seen. (b) Mild expansion of the upper mediastinum can be observed. A large pool of liquid and gaseous material is easily visible in the retrosternal area.

**Figure 4:**
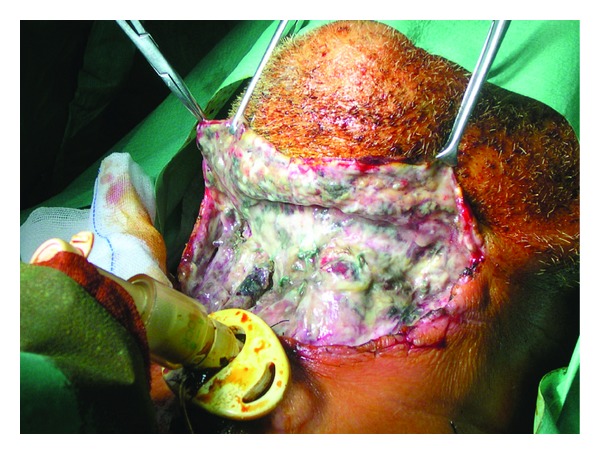
Patient number 2—surgical images. All the neck tissues appear necrotic and difficult to distinguish. Right septic thrombophlebitis of the internal jugular vein (Lemierre's syndrome) can be observed.

A 69-year-old diabetic male, suffering from a T1 glottic recurrence of squamous cell carcinoma previously treated with radiotherapy, was submitted to supracricoid laryngectomy without neck dissection. Perioperative and postoperative antibiotic therapy was routinely administered (amoxicillin plus clavulanate). During the very first postoperative days, the neck looked swollen and mildly painful without fever. The patient's general conditions appeared fairly good and the blood tests were in range (white blood cell count was within normal limits); on the fourth postoperative day a necrotic, anaesthetized area appeared immediately above the tracheotomy. The surgical exploration of the neck showed a diffuse necrotic area involving the anterior cervical area, above and around the laryngotracheal axis. Debridement of the necrotic tissues plus antibiotic lavages was performed, but the patient's condition did not improve and aggressive medical therapy (ciprofloxacin, meropenem, teicoplanin, metronidazole, low molecular weight heparin, cardiotonics, and proton pump inhibitors) became necessary. A CT scan of the neck revealed the presence of small pools of liquid/gaseous material in the neck planes, around the trachea and in the retrosternal area. No clear signs of neck abscesses were seen (Figure 3). At a second surgical exploration, all the neck tissues, including the muscles and partially the cartilages, appeared necrotic and difficult to distinguish. Right Lemierre's syndrome, a septic thrombophlebitis of the internal jugular vein, was observed (Figure 4). Moreover, necrotic secretions were seen even around the trachea in the superior thoracic inlet. An extensive debridement of the anterior cervical area and the neck spaces plus multiple antibiotic lavages (rifampicin) were carried out. The internal jugular vein was sacrificed and also a part of the neck musculature and the cutaneous flap. Multiple medications were performed every day and the patient was also submitted to hyperbaric oxygen therapy (HOT) for 8 days after the surgical procedure. Antibiotic and supporting therapy (metronidazole, meropenem, glycopeptides, and linezolid) were continued for other 50 days. Following this, a third, reconstructive surgical procedure was performed about 2 months afterwards in order to close the defect in the anterolateral part of the neck. His clinical conditions slowly improved and the patient could be discharged about three months later in good health and with minimal morbidity. Histologic analysis of the surgical specimen demonstrated massive and destructive necrotic areas involving the connective and the muscular tissues with aggressive signs of acute and chronic diffuse phlogosis. Microbiologic evaluation revealed a mixed flora: *Pseudomonas aeruginosa*, *Staphylococcus auricularis*, *Staphylococcus aureus*, and *Streptococcus acidominimus*.

## 3. Discussion

Necrotizing soft tissue infections are rapidly progressive multimicrobial infections of the soft tissues, usually involving the abdominal walls, perineum, limbs, and, much more rarely, the craniocervical region [7]. In these cases, the host organism is unable to circumscribe the infections and consequently abscesses do not develop. From a histologic point of view, necrosis can be observed in the connective tissue, with eventual invasion into the surrounding structures. In necrotizing craniocervical fasciitis there are usually areas of muscular necrosis, though not in a dominant manner. These conditions are often associated with an abuse of alcohol, heavy smoking, and precarious social and hygienic circumstances, as well as with immunodepressive or dysmetabolic conditions. Nonsteroidal anti-inflammatory drugs might be responsible for altering the immune system function (due to modification of granulocyte function), thus facilitating the development of NCSTI [8]. Nevertheless, this mechanism remains to be clarified and confirmed. While the second patient presented some risk factors, such as a dysmetabolic condition (diabetes mellitus), previous radiotherapy, and surgical injury, it is worthwhile pointing out that no risk factors were present in the first one and, furthermore, he had not been taking anti-inflammatory drugs or any other treatment. Thus, this aspect gives rise to several queries concerning the germ/host relationship, which, if modified, triggers this very severe condition.

The anatomical spread of infection from the craniocervical region to the thorax is well known [5]. Both of our cases presented a thoracomediastinal involvement and the thoracic surgeon was called to explore the mediastinum based on the degree of the thoracic involvement; patient number 1 was consequently debrided through a transcervical approach. In fact, transcervical drainage is recommended for invasion into the anterior and posterior mediastinum while transthoracic procedures are recommended in cases with greater invasion and in cases of massive pleural cavity involvement [5].

Early diagnosis is the key in treating this condition [9]. Delay in the treatment can lead to systemic toxicity that, in turn, leads to multiorgan deficiency. Furthermore, many complications can be associated with NCSTIs such as the obstruction of the upper airways, thrombosis of a large cervical vessel (Lemierre's syndrome), mediastinitis, pneumonia, and pleural effusion/empyema [10]. Usually, NCSTIs are aetiologically polymicrobial and many types of bacteria, both aerobic and anaerobic, have been isolated. The presence of such polymicrobism may be responsible for a sort of synergism that leads to an increased virulence of the infection and consequently to rapid necrosis of the tissues [3]. Polymicrobial flora was demonstrated in both our patients.

Diagnosis is mainly clinical; the simultaneous presence of systemic symptoms of sepsis (tachypnea, tachycardia, fever, and symptoms of deficiency in several organs) and local signs (oedema, swelling, and hyperaemia) should orient diagnosis towards NCSTI. The absence of pain can be justified by the damage of nerve terminals; neither of our patients complained of pain in the cervical area. An objective evaluation revealed signs of acute inflammation that would be normally recognized in a postoperative period. In fact, in patient number 2 there was a delay in diagnosis and consequently in treatment, due to the presence of this misleading factor. Only the appearance of a necrotic area in the anterior cervical region made us suspect what was happening. From a clinical point of view our patients were different: the first one was severely febrile while the second one had no fever. Moreover, signs of multiorgan failure rapidly appeared in the first one and the patient needed orotracheal intubation and intensive treatment. The infection in patient number 2 seemed to be less aggressive. In effect, this should lead to reflection; the white blood cell count was actually within normal limits, maybe demonstrating a poor defensive reaction. However, we believe that a strong clinical suspicion is crucial for an early and timely diagnosis.

CT is very important for the diagnosis as it can reveal the presence of liquid and gaseous materials between the cervical planes. In addition, increased thickness and enhancement of the perimuscular and subcutaneous soft tissues are often observed. Furthermore, CT has proven to be very important for detecting complications in the cervical and chest areas, too; in both of our patients these signs were easily revealed by the scans. Magnetic resonance, due to its greater sensitivity, offers the possibility of detecting the infection when it is limited to the cervical fascia, thus leading to the diagnosis of true craniocervical necrotizing fasciitis.

Delay in diagnosis is probably the most important factor that can affect the outcome, with the patient's general health conditions and the type of flora involved coming next; the mortality rate has been reported to be 15–20%, even if higher percentages have been reported in cases of mediastinal involvement [1, 2, 10, 11].

Aggressive treatment (both medical and surgical) is the key in the management of these patients. Antibiotic therapy should address almost all kinds of pathogens and should be targeted upon the antibiogram results; initially, however, an empirical use of large spectrum antibiotics is widely accepted [11]. Surgery should be aimed to excise the necrotic tissue through a large cervical flap. Bleeding healthy tissue should be reached at the end of the surgical exploration, even if it is not always an easy task. However, we want to stress the concept that surgical debridement must be performed as thoroughly as surgically possible and as aggressive as possible.

Hyperbaric oxygen therapy (HOT), via increasing phagocyte activity of neutrophils and inhibiting growth of anaerobic germs, seems to be helpful as a supplementary treatment [12, 13]. Furthermore, HOT has also been postulated as having a hypothetical role in the healing processes, due to the consequent increase in angiogenesis and deposition of collagen. Therefore, considering the dramatic situation of patient number 2 at surgery he was submitted to 8 dives during the postoperative period, which led to gradual improvement of the local situation. Furthermore, the application of fly maggots to devour the necrotic debris has been advocated as a nonconventional supporting therapy [14].

## 4. Summary

NCSTI is a rapidly progressive clinical condition, which, if not treated, can lead to sepsis and multiorgan deficiency. Early diagnosis and aggressive, multimodality treatment can reduce mortality and morbidity rates. Thoracic and mediastinal involvement requires appropriate management. Clinical suspicion remains one of the most important aspects in the management of such devastating conditions and treatment must be as aggressive as possible.
